# Response to: “Management of Chloroquine and Hydroxychloroquine Poisoning: Do Not Miss the Time of Tracheal Intubation and Mechanical Ventilation”

**DOI:** 10.5811/westjem.2020.10.50401

**Published:** 2021-01-11

**Authors:** Jacob A. Lebin, Kathy T. LeSaint

**Affiliations:** University of California San Francisco, Department of Emergency Medicine, San Francisco, California. California Poison Control System, San Francisco Division, San Francisco, California

To the Editor:

We thank Drs. Megarbane and Schicchi for their thoughtful comments on our manuscript and efforts to highlight pertinent in vitro and in vivo literature. As stated in our manuscript, we agree that aggressive supportive care is the mainstay of treatment for acute chloroquine and hydroxychloroquine toxicity, including management of the airway with appropriate ventilation, if necessary.

While Drs. Megarbane and Schicchi make a valid point on the importance of intubation and mechanical ventilation in patients with evidence of severe poisoning, the indications for early intubation prior to severe symptom onset are less clear. The writers suggest that intubation is required for any prognostic factor of death, such as a presumed ingestion of greater than four grams. In a retrospective case series of 167 patients with acute chloroquine poisoning, there was no correlation between the amount ingested by history and the peak blood chloroquine concentration; however, the peak blood chloroquine concentration was directly related to mortality, suggesting that the reported ingested dose has limited utility for predicting toxicity.^1^ Similarly, the writers comment that intubation is also required if the QRS duration is greater than 100 milliseconds (ms); but, of the 14 patient fatalities in that cohort, almost half had a QRS duration less than or equal to 100 ms.^1^ Therefore, suggesting that early intubation is required based on any single factor may result in misguided interventions without substantiated benefit. We recommend that intubation be considered based on clinician assessment of multiple factors, including severity of presenting symptoms and anticipated clinical course.

We also thank Drs. Megarbane and Schicchi for highlighting important in vitro and in vivo animal data regarding the utility of diazepam. The writers present data reporting that intravenous diazepam did not restore intrinsic mechanical performance in chloroquine-exposed rat cardiac papillary muscle or attenuate chloroquine-induced cardiotoxicity in poisoned rats.^2^,^3^ However, the later investigation also documents that the combined administration of diazepam and epinephrine did improve cardiac contractility.^3^ One important caution to consider for these data is the direct application of animal studies to human subjects, where ingested doses, symptomatology, and chronic toxicity may be variable. Thus, in patients with severe chloroquine or hydroxychloroquine poisoning who are mechanically ventilated, we believe it is reasonable to provide diazepam in addition to vasopressors and aggressive supportive care.

We encourage additional investigations examining the role and indications for intubation, mechanical ventilation, vasopressor support, and diazepam in the context of acute chloroquine and hydroxychloroquine poisoning.

## References

[1] Clemessy JL, Taboulet P, Hoffman JR (1996). Treatment of acute chloroquine poisoning: a 5-year experience. Crit Care Med.

[2] Riou B, Lecarpentier Y, Barriot P (1989). Diazepam does not improve the mechanical performance of rat cardiac papillary muscle exposed to chloroquine in vitro. Intensive Care Med.

[3] Hughes DA (2020). Acute chloroquine poisoning: a comprehensive experimental toxicology assessment of the role of diazepam. Br J Pharmacol.

